# Reply to “Comment on: Increases in arm volume predict lymphoedema and quality of life deficits after axillary surgery: a prospective cohort study”

**DOI:** 10.1038/s41416-021-01268-2

**Published:** 2021-03-17

**Authors:** Nigel Bundred, Chris Todd, Katie Riches, Vaughan Keeley, and the Investigators of BEA studies

**Affiliations:** 1 Manchester University NHS Foundation Trust, Manchester, UK; 2 University of Manchester, Manchester Academic Health Sciences Centre (MAHSC), Manchester, UK; 3 School of Health Sciences, Faculty of Biology, Medicine and Health, The University of Manchester, Manchester, UK; 4 University Hospitals of Derby and Burton NHS Foundation Trust, Derby, UK; 5 University of Nottingham Medical School, Nottingham, UK

**Keywords:** Translational research, Surgical oncology

We thank Boyages and colleagues for their comments^1^ on our paper.^2^

The ‘gold standard ‘of relative arm volume increase (RAVI) >10% defined the diagnostic criterion of lymphoedema in the study and not a threshold for early intervention.

There is no internationally agreed gold standard for the diagnosis of breast cancer related lymphoedema (BCRL) but at commencement of our study, an increase of 10% in volume of the affected arm compared with the unaffected arm was a commonly used criterion.^2,3^ Nevertheless, some patients develop clinical lymphoedema (hand or forearm swelling) with RAVI of <10%. Clinicians who felt that the patient had clinical lymphoedema with a RAVI <10% were permitted to initiate lymphoedema treatment. This produced a surrogate measure of clinical lymphoedema including both groups, but this definition only increased the percentage of women found to have lymphoedema from 22.8% to 24.5% at 24 months, a BCRL rate similar to that found in a large meta-analysis.^3^

The conventional definition of lymphoedema by bioimpedance (BIS) was an increase >10 units.^3–5^ Using this definition BCRL was present in 45.6% at 24 months. This is double the rate of clinical lymphoedema, indicating BIS overestimates the presence of lymphoedema and with a lower BIS threshold, this effect is even greater. A RAVI > 5% (and clinical symptoms) was a better criterion for early intervention but did not correlate well with BIS (although the percentage meeting this criterion was similar). Arm volume changes predicted symptom and lymphoedema development whereas BIS changes did not correlate particularly well with volume changes or subsequent lymphoedema.

Study participants received regular follow-up, so lymphoedema was identified and treated promptly before the development of fat and fibrosis deposition.

Another aim of the research was to define whether increase in BIS or RAVI would be a better predictor of the development of clinical lymphedema and select patients for screening and earlier treatment intervention. RAVI was the better predictor.

Amongst participants whose BIS change was >5 but <10 at 6 months post-surgery, only 19% went on to develop lymphoedema by 24 months (see Table 2) whereas for those with a RAVI 4–9% it was 43%.^2^ Of those with a BIS > 10 increase (BIS diagnostic criterion for lymphoedema) at 6 months only 33% of participants subsequently developed lymphoedema by 24 months, indicating that BIS is neither sensitive, specific nor predictive of lymphedema.^2^

Several other studies^3–5^ have found BIS changes do not predict lymphoedema.

The Quality of Life data showed that unless the individual has a RAVI >5% and clinical symptoms, applying a compression sleeve did not improve quality of life or prevent lymphedema.^2^

Evidence that early detection and intervention prevents clinical lymphoedema is poor. Recent randomised trials of early intervention with either manual lymphatic drainage or compression sleeves in women with RAVI 4–9% arm increases, found no benefit of either treatment on lymphoedema development.^6,7^ In the absence of effective interventions, the value of screening for a condition is debatable.

The main results focus on the follow-up at 24 months which is the time by which >90% of BCRL develops. The data presented does not include all follow-up to 5 years as the data census was carried out before all participants had reached this stage. The percentage with data available at 24 months was 49.5%. Inevitably, the percentage of participants still being followed at five years will be lower. However, we believe that the numbers at each time point are sufficiently large and this does not affect the main study findings.

Our results conflict with the interim analysis of the PREVENT study.^8^ Fewer patients in the BIS group triggered an intervention compared with patients in the tape measure (TM) group (15.8% (BIS) vs. 28.5% (TM)).

Since this occurred in the absence of any previous intervention, the groups may not have been comparable at baseline. The differences may reflect differences in thresholds to trigger, and not necessarily that BIS is superior to volume measurements.^8^ It is recognised that volume measurements by TM are less accurate and more subject to inter-rater variation than Perometer measurements. This is why we chose Perometry and all those carrying out the measurements received standardised training by an experienced researcher. The findings in the PREVENT study were preliminary and nonsignificant statistically and thus have no clinical significance.

In our large multicentre screening study, BIS overestimated the incidence of BCRL^2,9,10^ and its routine use alone for screening (or sleeve application) would lead to over diagnosis and overtreatment of lymphedema.

## Data Availability

The data and material are all available through writing to Manchester CTU (formerly MAHSCCTU).
